# Oropouche Virus–Associated Aseptic Meningoencephalitis, Southeastern Brazil

**DOI:** 10.3201/eid2502.181189

**Published:** 2019-02

**Authors:** Sebastian Vernal, Camila C.R. Martini, Benedito A.L. da Fonseca

**Affiliations:** University of São Paulo, Ribeirão Preto, Brazil

**Keywords:** Oropouche virus, Oropouche fever, arboviruses, vector-borne infections, viruses, Brazil, mosquitoes, tropical diseases, arthropod-borne diseases, zoonoses

## Abstract

Oropouche fever is a neglected arthropodborne disease and zoonosis responsible for several outbreaks of a febrile disease in Central and South America. We present a clinical case of aseptic meningoencephalitis in an immunocompetent patient that resulted from Oropouche virus acquired in northern Brazil but diagnosed in a nonendemic region.

In April 2016, a 28-year-old man (S.V.) in Ribeirão Preto, southeastern Brazil, with a history of Gilbert syndrome sought care for a sudden high fever, severe 1-sided headache, vomiting, intense photophobia, stiff neck, and confusion. Seventeen days earlier, he had returned from a trip to Mosqueiro Island in northern Brazil; he received yellow fever vaccine 5 days before traveling. His 1-week visit to the island included outdoor activities that involved entrances into the native Amazon rainforest. The patient returned to Ribeirão Preto and remained asymptomatic for 10 days before becoming acutely ill with fever, chills, myalgia, headache, and dizziness. Symptoms occurred intermittently for 3 days, which prompted him to seek medical care. After evaluation, a diagnosis of dengue fever was considered. Microhematocrit was 54%, and tourniquet test was negative. Nonstructural protein (NS) 1 antigen detection test was requested, and the patient was treated with medications for his symptoms and prescribed abundant oral hydration and bed rest. Dengue fever was ruled out because NS1 antigen detection and IgM serologic testing provided negative results 6 days after initial symptom onset. Therefore, he was referred to an infectious disease outpatient clinic at another healthcare facility for further investigation. Yellow fever vaccine reaction and malaria were initially the main hypotheses, but thick blood film examination results were negative.

Seven days after initial symptom onset, the patient’s headache worsened and became left-sided and pulsatile; intense photophobia, vomiting, and fever (105.8°F) developed. No hemorrhagic manifestations were observed. He was admitted to the emergency department at the Teaching Hospital in Ribeirão Preto with nuchal rigidity; ceftriaxone was initiated.

At admission, he was confused, including attention deficits and hallucinations. Physical examination found tachypnea (27 breaths/min), heart rate 76 beats/min, and blood pressure 130/80 mm Hg; cardiopulmonary examination results were unremarkable, and the liver was palpable at 3 cm under the costal margin (spleen was not palpable) with no pain at abdominal palpation. The patient exhibited mild spasticity of lower limbs with positive Babinski sign, without focalities at neurologic exam. Because of clinical examination findings, viral meningoencephalitis emerged as a possible diagnosis, and intravenous acyclovir was started.

Laboratory examination showed complete blood counts within reference ranges (hematocrit 44%; leukocytes 10,300 cells/mm^3^ [no left shift; lymphocytes 2,300 cells/mm^3^]; platelets 388,000/mm^3^), normal kidney function, and sodium and potassium concentrations within reference ranges. Liver enzymes were slightly elevated (aspartate aminotransferase 30 U/L [reference 15–46 U/L], alanine aminotransferase 71 U/L [reference 13–69 U/L], γ-glutamyl transferase 107 U/L [7–60 U/L]); total bilirubin was 0.73 mg/dL (reference 0.2–1.0 mg/dL). Serologic test results were nonreactive for hepatitis A, B, and C; HIV; syphilis; Epstein-Barr virus; *Toxoplasma gondii*; and *Trypanosoma cruzi*. Thorax radiograph imaging was unremarkable; cerebral computed tomography scan showed cortical edema on the left frontal lobe (Figure, panel A).

**Figure:**
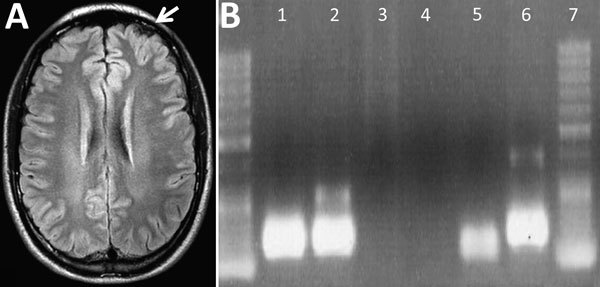
Imaging and PCR results for a 28-year-old man with Oropouche virus infection, southeastern Brazil. A) Cerebral computed tomography showing a cortical edema on the left frontal lobe (white arrow). B) Agarose gel of reverse transcription PCR products of Oropouche virus S fraction. Lane 1, patient’s serum sample; lane 2, patient’s cerebrospinal fluid; lane 3, patient’s leukocyte supernatant; lane 4, negative control; lane 5, internal control; lane 6, positive control.

Results of a spinal tap showed a 23 cm H_2_O opening pressure (reference 5–20 cm H_2_O) with clear cerebrospinal fluid containing 45 cells/mm^3^ (reference <5 cells/mm^3^) (85% lymphocytes); protein concentration of 53 mg/dL (reference <45 mg/dL); and glucose concentration of 62 mg/dL (capillary 100 mg/dL [reference 2/3 of capillary glucose]). VDRL test results were negative, and bacterial and fungal antigens were not detected. PCR results were negative for enterovirus, herpes viruses 1 and 2, varicella zoster virus, cysticercosis, tuberculosis, and toxoplasmosis but positive for Oropouche virus (OROV) (fraction S) (*1*). Blood sample PCR results were also positive for OROV (Figure, panel B) (*2*), and indirect immunofluorescence antibody testing (Appendix) (*3*) confirmed OROV infection.

After 7 days of hospitalization, the patient recovered all neurologic function with no sequelae (posttreatment electromyography and magnetic resonance imaging findings were unremarkable). He was discharged and has had no additional relapses.

OROV, an RNA virus belonging to the genus *Orthobunyavirus*, family Peribunyaviridae, is the causative agent of Oropouche fever in humans (*4*). In a sylvatic cycle, OROV can be transmitted to some animals by mosquitoes and in an urban environment can be transmitted to humans by the midge *Culicoides paraensis* (*5*). Usually, humans become infected in forested areas and then can translocate the virus to an urban environment (*6*).

After a 4- to 8-day incubation period, fever, headache, myalgia, arthralgia, chills, photophobia, dizziness, nausea, and vomiting develop (*2*,*6*). Less frequently, patients experience rash, anorexia, retro-orbital pain, and general malaise (*2*,*6*,*7*). Hemorrhagic phenomena also have been described (*8*). Most patients recover spontaneously after 7 days, although some experience symptoms for as long as 1 month (*2*,*6*,*7*). Some cases can relapse after recovery; the clinical picture is similar to the initial onset or can be more severe, including aseptic meningitis (*9*,*10*). These patients may experience neck stiffness, dizziness, vomiting, lethargy, diplopia, and nystagmus, but prognosis without sequel is usually good (*10*).

There has been an increased concern that Oropouche fever, endemic in northern Brazil, might spread across the country by contiguous urban cycles and by human movement. Physicians working worldwide in areas to which OROV is not endemic should include this neglected disease in the differential diagnosis of acute febrile syndrome, especially in patients visiting high-risk areas for OROV transmission.

Appendix Indirect immunofluorescence antibody test results for a 28-year-old man with Oropouche virus infection, southeastern Brazil.

## References

[1] Cardoso BF, Serra OP, Heinen LB, Zuchi N, Souza VC, Naveca FG, et al. Detection of Oropouche virus segment S in patients and in*Culex quinquefasciatus* in the state of Mato Grosso, Brazil. Mem Inst Oswaldo Cruz. 2015;110:745–54. 10.1590/0074-02760150123 26517653 PMC4667577

[2] Travassos da Rosa JF, de Souza WM, Pinheiro FP, Figueiredo ML, Cardoso JF, Acrani GO, et al. Oropouche virus: clinical, epidemiological, and molecular aspects of a neglected orthobunyavirus. Am J Trop Med Hyg. 2017;96:1019–30. 28167595 10.4269/ajtmh.16-0672 PMC5417190

[3] de Souza Luna LK, Rodrigues AH, Santos RI, Sesti-Costa R, Criado MF, Martins RB, et al. Oropouche virus is detected in peripheral blood leukocytes from patients. J Med Virol. 2017;89:1108–11. 10.1002/jmv.24722 27787907

[4] Vasconcelos PF, Calisher CH. Emergence of human arboviral diseases in the Americas, 2000–2016. Vector Borne Zoonotic Dis. 2016;16:295–301. 10.1089/vbz.2016.1952 26991057

[5] Pinheiro FP, Hoch AL, Gomes ML, Roberts DR. Oropouche virus. IV. Laboratory transmission by *Culicoides paraensis.* Am J Trop Med Hyg. 1981;30:172–6. 10.4269/ajtmh.1981.30.172 7212164

[6] Romero-Alvarez D, Escobar LE. Oropouche fever, an emergent disease from the Americas. Microbes Infect. 2018;20:135–46. 10.1016/j.micinf.2017.11.013 29247710

[7] Vasconcelos PF, Travassos Da Rosa JF, Guerreiro SC, Dégallier N, Travassos Da Rosa ES, Travassos Da Rosa AP. [1st register of an epidemic caused by Oropouche virus in the states of Maranhão and Goiás, Brazil] [in Portuguese]. Rev Inst Med Trop Sao Paulo. 1989;31:271–8. 10.1590/S0036-46651989000400011 2516642

[8] Alvarez-Falconi PP, Ríos Ruiz BA. [Oropuche fever outbreak in Bagazan, San Martin, Peru: epidemiological evaluation, gastrointestinal and hemorrhagic manifestations] [in Spanish]. Rev Gastroenterol Peru. 2010;30:334–40. 21263761

[9] Bastos MS, Lessa N, Naveca FG, Monte RL, Braga WS, Figueiredo LT, et al. Detection of Herpesvirus, Enterovirus, and Arbovirus infection in patients with suspected central nervous system viral infection in the Western Brazilian Amazon. J Med Virol. 2014;86:1522–7. 10.1002/jmv.23953 24760682

[10] Pinheiro FP, Rocha AG, Freitas RB, Ohana BA, Travassos da Rosa AP, Rogério JS, et al. [Meningitis associated with Oropouche virus infections] [in Portuguese]. Rev Inst Med Trop Sao Paulo. 1982;24:246–51. 6818668

