# Supplementary material for: Oropouche Virus–Associated Aseptic Meningoencephalitis, Southeastern Brazil
Source: Emerg Infect Dis. 2019 Feb;25(2):380–2. doi: 10.3201/eid2502.181189 (PMC6346467; doi:10.3201/eid2502.181189)
Supplement: Appendix — Indirect immunofluorescence antibody test results for a 28-year-old man with Oropouche virus infection, southeastern Brazil. [file 18-1189-Techapp-s1.pdf]

Article DOI: https://doi.org/10.3201/eid2502.181189

Oropouche Virus–Associated Aseptic Meningoencephalitis, Southeastern Brazil

Appendix

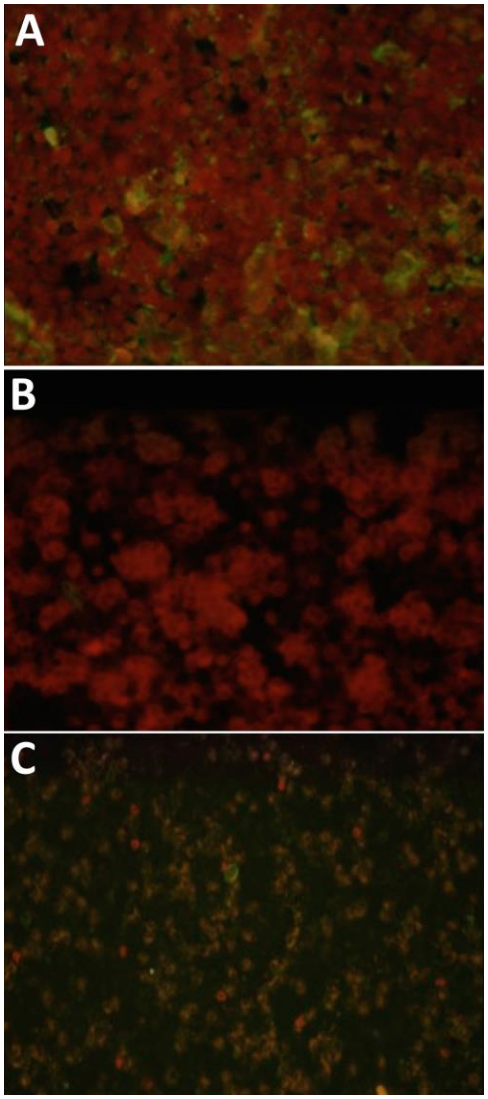

Appendix Figure. Indirect immunofluorescence (IIF) antibody test results for a 28-year-old man with Oropouche virus (OROV) infection, southeastern Brazil. IIF was performed using anti-human IgG conjugated with Fluorescein isothiocyanate. A) Vero cells and anti-OROV serum (negative control). B) Vero cells plus OROV and anti-OROV serum (positive control). C) Patient’s serum sample and anti-OROV serum. Original magnification ×40.
